# Response Time, Visual Search Strategy, and Anticipatory Skills in Volleyball Players

**DOI:** 10.1155/2014/189268

**Published:** 2014-04-30

**Authors:** Alessandro Piras, Roberto Lobietti, Salvatore Squatrito

**Affiliations:** ^1^Department of Biomedical and Neuromotor Sciences, Section of Human and General Physiology, University of Bologna, 40126 Bologna, Italy; ^2^Department of Histology, Embryology and Applied Biology, University of Bologna, 40126 Bologna, Italy

## Abstract

This paper aimed at comparing expert and novice volleyball players in a visuomotor task using realistic stimuli. Videos of a volleyball setter performing offensive action were presented to participants, while their eye movements were recorded by a head-mounted video based eye tracker. Participants were asked to foresee the direction (forward or backward) of the setter's toss by pressing one of two keys. Key-press response time, response accuracy, and gaze behaviour were measured from the first frame showing the setter's hand-ball contact to the button pressed by the participants. Experts were faster and more accurate in predicting the direction of the setting than novices, showing accurate predictions when they used a search strategy involving fewer fixations of longer duration, as well as spending less time in fixating all display areas from which they extract critical information for the judgment. These results are consistent with the view that superior performance in experts is due to their ability to efficiently encode domain-specific information that is relevant to the task.

## 1. Introduction

Sport expertise has been defined as the ability to consistently demonstrate superior athletic performance. It is generally accepted that expert athletes perform better than novices but it is not clear whether superior performance stems from more refined sensory-motor coordination alone. Athletes must be able to identify the most information-rich areas of the visual field, direct their attention appropriately, and extract meaning from these areas efficiently and effectively [1]. In a successful sport performance, knowing where and when to look may be crucial, especially when the scene is wide and information relevant to the task can be presented in different forms and locations.

Athlete's superiority on beginners in elementary visuomotor tasks, such as visual acuity, saccadic eye movements, depth perception, and oculomotor reaction time, has been widely investigated but results cannot be generalized to all sports [2–5]. As such, the differences in visual search strategy, that is, the ability to quickly locate task-relevant information between expert and novice players, in one sport may be inconsistent with those of others [6]. Anticipation is an important part in sports expertise; it refers to the ability to predict what is likely to happen prior to the event itself. This ability to “read the play” is essential in sport where the speed of the game means that decisions must typically be made in advance of an opponent's action. Key factors behind anticipation in sport include visual abilities and perceptual and cognitive skills. The ability to recall and recognise an evolving pattern of play is the strongest predictor of anticipatory skill in team ball sports. For instance, we cannot compare anticipation of a volleyball serve, where the main goal is to send the ball over the net into the opposing court, with the anticipating of a volleyball setting action, where the purpose is to set the ball in proper position for the attack. The task in the first case may require different information processing strategies with respect to the other, such as the detection of postural cues or the recognition of an evolving pattern of play. Therefore, in certain situations it is conceivable that players may rely exclusively on the ability to process information from an opponent's postural orientation, whereas in others they have to make an anticipatory judgment based on perceived event probabilities. The ability to extract important information from a sport action, even in the same sport, may be related to the type of action being examined.

In this research we addressed the question whether gaze behaviour can affect performance in volleyball. In this regard, volleyball has been extensively investigated, but we still do not have exhaustive results that let us know about better visual search strategy of expert over novice players. Some authors, using simple laboratory tasks, demonstrated that there are specific visual skills and better results in advanced volleyball players compared with beginners [7–9]; others showed faster simple reaction times (RTs) in expert than novice players [10–12]; still others established some differences in gaze strategy due to the level of expertise [13–15]; and a few studies have reported athletes performing a little faster than novices, in the choice of RT tasks with generic stimuli [11, 16].

In volleyball, experts perform better in tasks concerning perceptual speed, extent of the focus of attention, prediction and estimation of speed, and direction of moving objects [13, 17]. Relevant information would include, among others, the ball distance, its angle, velocity, and acceleration of descent, setter's body movements, as well as the appropriate procedures for using these variables to correctly evaluate the ball trajectory. What processes volleyball player actually can and do use to solve the task? As a result, employing a strategy that accurately and swiftly identifies and interprets the relevant information from the surroundings ensures that athletes are better prepared to perform successfully. As it turns out, human decision makers often use simple rules that neither require all available relevant information nor integrate the information that is used but that however allows them to accomplish their aims quickly and effectively, following a simple heuristic [18]. Shim et al. [19] found that increasing the information shown to the participants had opposite effects on novice and skilled performers. Novice players' anticipation accuracy decreased as more information was presented because they are unable to distinguish between relevant and irrelevant information.

Piras et al. [13] compared gaze strategy of expert and novice volleyball players while observing a filmed action in which the coach tosses the ball to the setter. They found that expert players did a fewer number of fixations of longer duration to the setter's hands and body, likely trying to gain the greatest information from the body motion, to predict the ball trajectory. This simple decision strategy, also called simple heuristic [20], can rely on the concept that less (in terms of fewer number of fixations) can be more (in terms of predictive ability) connected to the idea that athletes use less information or require fewer cognitive steps to achieve the target [21]. From the simple heuristic perspective, visual search strategy is based on the importance of the cues and their relationships as a simple decision strategy for future actions. The ability to extract better quality information per fixation and to acquire information more effectively via peripheral vision contributes to expert's superior anticipation in these contexts. By contrast, novices jump back and forth between relevant and irrelevant regions, probably because they cannot distinguish task-relevant from task-irrelevant cues, thus producing many fixations of shorter durations. If one cue is more important than the sum of the others, experts can use this knowledge, which they gain from experience, and stop searching after considering one cue. On the other hand, novices consider multiple cues because they have not figured out which one is the most predictive and they have more problems to distinguish which cue is important when the amount to be processed increases (for a review see [20]). In line with the simple heuristics explanation, some authors reported that experts show fewer fixations of longer durations within the task [22]. However, other studies have found an opposite results pattern, with experts showing an increased number of fixations of shorter duration on more cues than novices [23]. These divergent results indicate that the simple heuristics explanation cannot completely account for expert's superior performance.

To explain the shorter fixation durations of experts, a long-term memory hypothesis has been proposed. According to this hypothesis, experts can encode and retrieve information more rapidly than novices and thus have on average shorter fixation durations than novices. Long-term working memory is a memory skill that individuals acquire to meet the particular memory demands of a complex cognitive activity in a particular domain. In order to attain skilled performance, individuals acquire domain-specific knowledge, procedures, and various perceptual motor skills. To meet the particular demands for working memory in a given skilled activity, subjects acquire encoding methods and retrieval structures that allow efficient storage and retrieval from long-term memory (for a review see [24]). According to the long-term memory hypothesis, if expertise in a particular context is based on more efficient encoding and retrieval of information, we can expect fixations of shorter durations. What differs is the point of view of the information reduction hypothesis [25] that focuses on the learned selectivity of information processing. Experts optimize the amount of processed information focusing on task-relevant information and arrive at more accurate predictions. This is accomplished through strategic considerations in order to selectively allocate attentional resources. Therefore, experts should exhibit fewer fixations of shorter duration on task-redundant areas and more fixations of longer duration on task-relevant areas. Previous studies have shown that athletes focus their attention on task-relevant areas and overall show fewer fixations of longer durations than novices, presumably because experts use parafoveal regions to extract information from a large number of visual cues [13, 26, 27]. These results seem inconsistent with the long-term memory hypothesis, which would have predicted shorter fixation durations for experts.

In a previous study [13] we presented a viewing task where experts and novices were instructed to watch video clips very carefully. The task was different with respect to the present study because the video clips showed the complete sequence of setter's action with the ball tossed to either forward (to field position 4) or backward (to field position 2). Our first intention was to discover the differences in visual search strategy analyzing the mean number and duration of each fixation, identifying where, when, and how athletes fixate their gaze in order to better understand opponent's tactical behaviour. This second purpose was achieved by analyzing the temporal sequence of fixations, that is, a quantitative analysis of the most frequent gaze shifts over the different interest areas. Results favoured an information reduction account, showing that expert volleyball players, compared to novices, used a search strategy involving fewer fixations with longer duration, spending more time fixating first at the initial ball flight and then making a saccade to setter's hand, disregarding the intermediate phase of the ball trajectory. Their gaze was directed mainly to setter's body [13]. A shortcoming of this study was that the key-press response time and the response accuracy were not considered.

In order to study the relationship between gaze strategy and anticipatory processes, in this paper we report results of key-press response time and gaze behaviour recorded from experts and novices, in a task in which the participants were required to predict the target location of setter's toss. According to previous research [13], we wanted to test the hypothesis whether expert volleyball players demonstrate superior anticipation, defined as higher response accuracy and shorter key-press response times, with respect to novices and, if so, whether this superiority can be explained with the use of a more efficient and effective visual search strategy, evaluated as fewer number of fixations of longer durations on relevant interest areas.

Furthermore, given that the majority of researchers interested in visual search behaviour in sports, and particularly in volleyball, have attempted to identify differences in point of gaze as a function of expertise, it is important to examine whether successful performers employ different visual search patterns than unsuccessful performers within a group where the participants are presumed to have a similar level of expertise [28]. Several researchers have highlighted the potential advantage of using a within-task criterion to stratify participants into groups, to reduce variability in performance level in measures of perceptual-cognitive skill [28–30]. Thus, to strengthen the hypothesis of a relationship between gaze strategy and action effectiveness, we wanted to determine whether there are any differences in visual search behaviour within a group of experts on successful versus unsuccessful volleyball settings. This methodology would increase measurement sensitivity and highlight the potential relationship between visual search behaviour and decision making skill in simulated dynamic volleyball setting action.

## 2. Methods

### 2.1. Participants

Fifteen expert volleyball players (Italian professionals league B1) and fifteen novices were recruited for the study. Novices had not participated in any sport at a professional level, and although they all knew the rules and the practice of volleyball, they had never participated regularly in volleyball. Mean age of participants was 24.47 ± 1.52 years (experts = 24.87 ± 1.92; novices = 24.07 ± 0.88, *F* = 2.14, *P* = 0.15, Cohen's *d* = −0.15, *r* = −0.26). They all voluntarily underwent the test, which did not include any invasive or harmful procedures. All participants received a verbal explanation of experimental procedures and gave their written informed consent before participating in the study. None of them reported any uncompensated visual deficit or difficulty with the stimuli used in the present study. The experimental protocol was approved by the Institutional Ethic Committee of the University of Bologna.

### 2.2. Test Film

**Figure 1:**
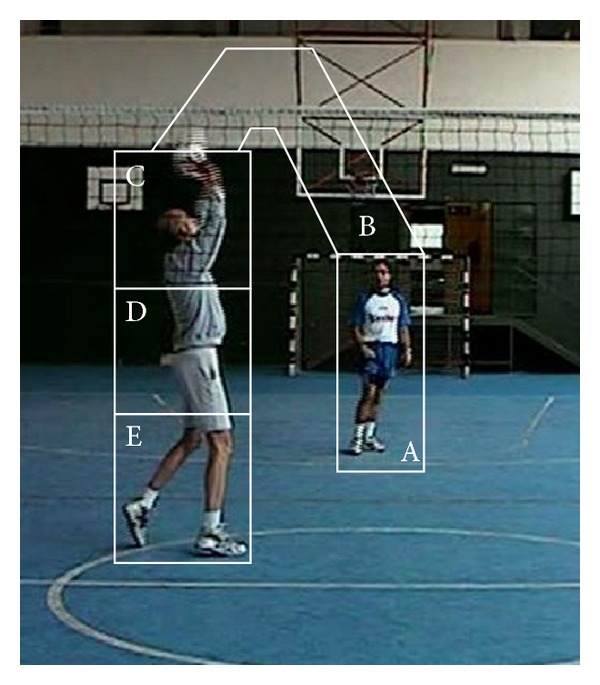
Viewpoint of the participant showing the setter's hands-ball contact. White lines delineate the interest areas. A = IA-1, coach; B = IA-2, ball trajectory; C = IA-3, setter's hands; D = IA-4, setter's trunk; and E = IA-5, setter's legs. Areas external to the contours are considered as out.

The setup was the same used in a previous research [13]. A setter was filmed from the block and defense team's perspective. The film clips were recorded using a digital video camera (Hitachi Dz-Mv270 e) at 30 frames/s, with resolution of 1280 × 960 pixels, and placed 154 cm from the floor and 550 cm from the net in the middle of the court. The filmed action consisted of the coach (positioned on back zone of the volleyball court) tossing the ball to the setter (positioned on front zone of the volleyball court), who had to set it either forward (to field position 4) or backward (to field position 2). When presented to the experimental participants the film finished when the ball touched setter's hands (see Figure 1). Specifically, we stopped the film sequences at the moment when the setter received the ball to prevent participants from receiving any feedback in relation to the decision that was made during the actual filming session.

The filming perspective we used provided a wide viewing angle and some perspective, which enable us to facilitate the perception of depth. That viewing angle provided the closest correspondence to the field of view that a central defensive player typically observes. We therefore asked the participants to image themselves as a defensive blocker playing in a central position that was just in front of the camera. The setter had to play as if he was in a real game, doing the perfect pass for the hitter. The role of the blocker is to read opponent's setter and determine where the ball will be sent, and once the ball is hit, he/she has to try to block it.

Twelve 6 sec video clips, containing either a forward or a backward set, were shown to each subject for 10 times in a random order. Thus, each participant viewed a total of 120 trials.

### 2.3. Apparatus

**Figure 2:**
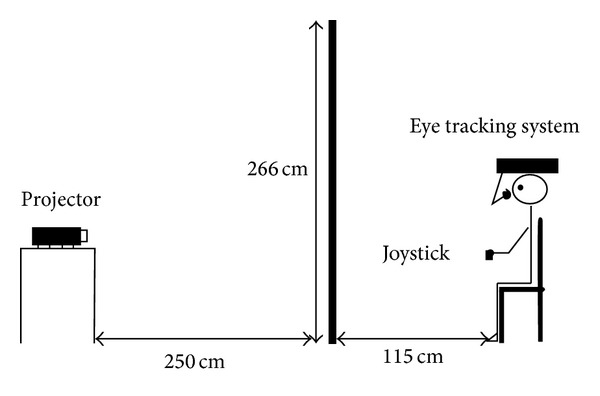
A lateral view of the experimental setup.

The participants sat on a chair, in front of a vertical translucent screen (266 × 269 cm), 115 cm from subject's eyes (see Figure 2). The sequences were back-projected by a digital projector with a resolution of 1024 × 768 pixels, distant 250 cm from the screen and forming an image 87 cm high and 120 cm wide. Eye movements were recorded binocularly by a video-based eye tracking system (EyeLink II, SR Research Ltd, Mississauga, Canada). The system consists of two miniature cameras mounted on a leather-padded headband. The entire system, weighing ~420 g, has a low center of mass for stability and subjective comfort. Pupil tracking was performed at 500 samples/s, with a gaze resolution <0,005° and noise limited to <0.01°. Data were encoded by software (Eyelink Data Viewer) that allows displaying, filtering, and presenting the results. Only data regarding the right eye were analyzed for this work. Gaze behaviour consisted of gaze fixations defined as the time the eye remained stationary (within 1.5° window) for a period greater than 99.99 ms. All events corresponding to eyelid occlusion (blinks), to pupil size very small, or to image missed or severely distorted were discarded. Events that occurred 100 ms before or after a blink were also discarded.

### 2.4. Procedure

Before each participant was tested, eye tracking calibration was carried out, in order to link participant's eye position to specific positions on the screen. To do this, eye position was recorded while randomly presenting a target on regularly spaced points of a nine-point grid of known size; then, data validation was performed after each block of twelve videos. Finally, drift correction was executed after each trial.

In the video stimulus, each video clip started with a 500 ms acoustic tone to prepare participants for the video onset. The duration of each clip was equal across all trials. The participants were required to follow the action as if they were on volleyball field. The locations of the volleyball sets were completely randomized but kept in the same order for each participant. The filmed action consisted of the coach tossing the ball to the setter, who had to set either forward (to court position 4) or backward (to court position 2). When the ball reached setter's hands, the participant had to determine as quickly and accurately as possible whether the offensive action in the stimulus would come forward or backward of the setter. The response was given by pressing one of two buttons (right or left) of a game pad. The response cleared the screen (i.e., caused the end of the video clip) for the next video to begin. No feedback was given to the participants as to their performance on each trial. Practice, calibration, validation, rest periods, and data collection took on average 30–40 minutes per participant. Practice (5 similar trials but with different video clip) was necessary in order to avoid incorrect response due to the lack of ability to use the system. Participants were encouraged to take a break of 5 minutes midway through the experiment, after 60 trials.

### 2.5. Dependent Variables and Analysis

*Anticipation Test*. The ability to make accurate predictions from advanced sources of information was measured as follows.

(i)* Response Accuracy*. The percentage of trials in which subject's response was correct or incorrect (i.e., forward or backward judgement) was determined.

(ii)* Key-Press Response Time*. The time (ms) from setter's hands-ball contact to the button pressed by the participant was determined. Responses with RTs shorter than 150 msec and longer than 600 msec were discarded (early or delayed responses) [11]. After preprocessing we used 980 videos for athletes (from 1800 or 54%, 820 videos were excluded) and 669 for novices (from 1800 or 37%, 1131 videos were excluded). Response time was also related to the accuracy of the response (correct/incorrect).

*Visual Search Data*. The following visual search measures were analyzed.

(i)* Search Rate*. This measure included the mean number of visual fixations and the mean fixation duration per trial between groups across correct and incorrect responses.

(ii)* Percentage Viewing Time*. Mean percentage of time participants spent fixating the gaze on each interest area of the display when trying to anticipate ball direction was determined. For this purpose, the screen was divided into five areas (IAs): (i) IA-1, 6° (width) × 12° (height) of visual angle in size, included the coach that performs the pass to the setter; (ii) IA-2, 10° (w) × 20° (h), included the ball trajectory from the coach to the setter; and (iii) IA-3 to IA-5 subdivided the setter's body in three: the first part, 12° (w) × 6° (h), includes the hands and the shoulders, the second part, 12° (w) × 6° (h), includes the body from the shoulders to the hip, and the third one, 12° (w) × 8° (h), is from the hip to the tip of the feet. All fixations outside these IAs were referred to as “out” fixations (see Figure 1).

We analyzed these data in relation to key-press response time and accuracy of the response (correct, incorrect). To examine whether performance levels changed across consecutive sessions, repeated measures analyses of variance (ANOVA) were carried out for each variable, with trial (10) as the within-subject factor. Mauchly's test was considered for each variable to assess assumptions of sphericity. If assumptions of sphericity were violated, the Greenhouse-Geisser epsilon corrections of degrees of freedom were used [31].

## 3. Results

No within-session practice effects were observed for any variable; the reason might be related to the lack of feedback in the study.

### 3.1. Anticipation Test

The anticipation test variables are presented in Table 1.

A repeated measures ANOVA was conducted on the proportion of correct responses in which setting directions (backward, forward) were the within-subjects factors and expertise (experts, novices) the between-subjects factor. ANOVA showed a significant main effect for expertise (*F*_1,28_ = 5.50, *P* = .026, and *ηp* ^2^ = .16), suggesting that participants showed more correct responses in comparison to novices (see Table 1).

A 2 × 2 repeated measures ANOVA was also done to analyse the key-press response time in which setting direction (backward, forward) and response accuracy (correct, incorrect) were the within-subjects factors, expertise (experts, novices) the between-subjects factor. ANOVA showed a significant main effect for expertise (*F*_1,28_ = 5.20, *P* = .038, and *ηp* ^2^ = .13) and a response accuracy × expertise interaction effect (*F*_1,28_ = 5.22, *P* = .030, and *ηp* ^2^ = .15). The main effect of expertise was due to the fact that experts showed a shorter key-press response time with respect to novices. The interaction effect was due to the fact that experts took longer to provide correct than incorrect responses, whereas novices showed the opposite results pattern, of longer response times on incorrect trials (see Table 1).

The results showed a clear effect of expertise, with shorter RT and higher accuracy for experts than novices (91% versus 77%).

### 3.2. Visual Search Data

All search rate dependent variables (number of fixations and fixation durations) were analysed separately using a 2 × 2 repeated measures ANOVA, with response accuracy (correct, incorrect) and setting direction (backward, forward) as a within-subjects factor, and expertise (experts, novices) as a between-subjects factor.

Analysis of mean fixation durations showed significant main effects for expertise (*F*_1,28_ = 25.29, *P* < .001, and *ηp* ^2^ = .47) and for expertise × response accuracy interaction (*F*_1,28_ = 4.29, *P* = .048, and *ηp* ^2^ = .11).

Analysis of mean number of fixations also showed significant main effects for expertise (*F*_1,28_ = 62.66, *P* < .001, and *ηp* ^2^ = .69) and for expertise × response accuracy interaction (*F*_1,28_ = 36.90, *P* < .001, and *ηp* ^2^ = .56).

Experts performance and correct responses were both associated with a lower number of fixations and shorter fixation durations. Athletes made 12.72 and 15.62 number of fixations with 508.50 and 495.27 milliseconds of fixation durations for correct and incorrect responses. Novices made 20.61 and 17.12 number of fixations with 444.22 and 466.75 milliseconds of fixation durations for correct and incorrect responses, respectively.

### 3.3. Correlation Analysis

**Figure 3:**
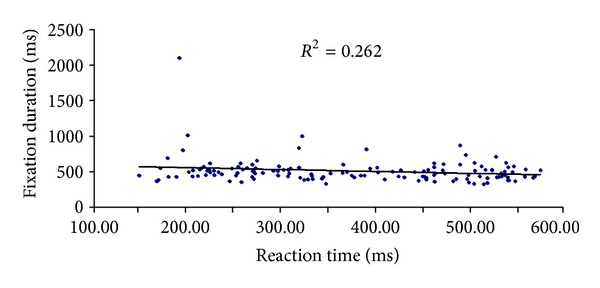
Correlation between gaze parameter and response time in experts' correct responses. Dots represent all participants' mean value for each correct trial. The line represents the regression coefficient (*r* = −.22,  *P* < .001) for expert groups.

To assess whether the pattern of response time correlates with gaze behaviour, we calculated the correlation between gaze parameters and response time in correct and incorrect responses across groups. The partial correlations procedure computes partial correlation coefficients that describe the linear relationship between two variables while controlling for the effects of one or more additional variables (*P* < 0.05). There was a significant correlation between fixation durations and response time in expert's correct responses when fixation number was a control variable (*r* = −.22, *P* < .001) (see Figure 3). A significant correlation was also found between fixation durations and number of fixations in experts (*r* = −.18, *P* = .003) and novices' correct responses (*r* = −.19, *P* = .003) using response time as a control variable. None of the corresponding correlations were significant for incorrect responses.

### 3.4. Percentage Viewing Time

**Figure 4:**
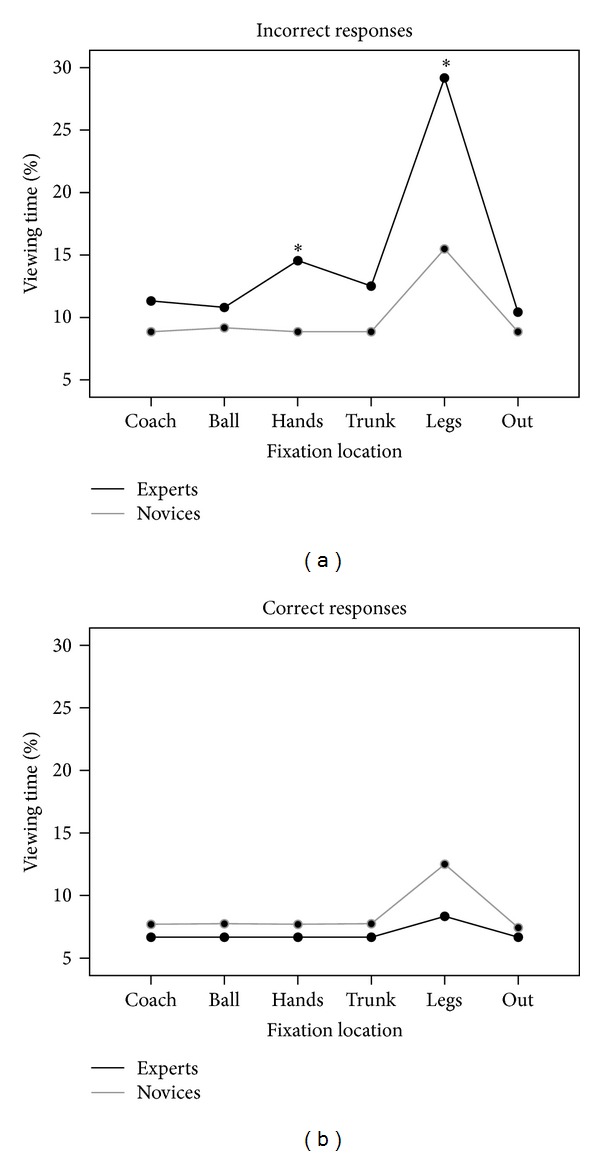
Mean percentage of time spent by subjects viewing each fixation location for the correct and incorrect responses across groups. Black lines = experts; gray lines = novices. Asterisks show significant differences (*P* < .001).

**Figure 5:**
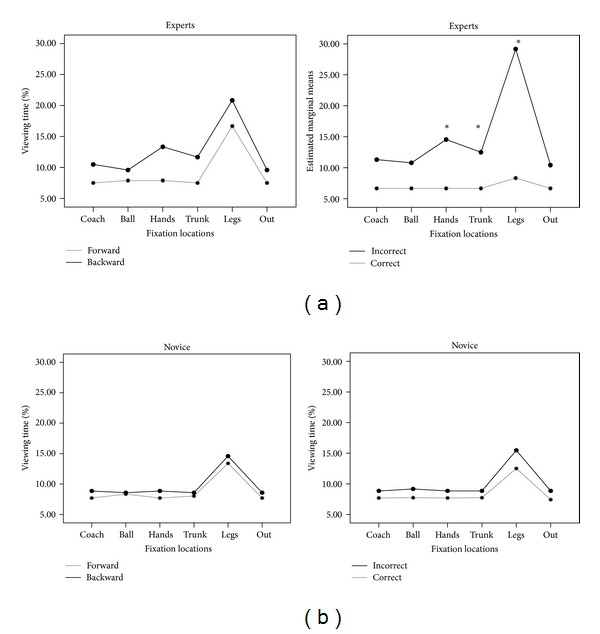
Mean percentage of time spent by experts (a) and novices (b) viewing each location for the backward (black lines) and forward (gray lines) settings on the left chart and incorrect (black lines) and correct (gray lines) responses to the right chart. Asterisks show significant differences (*P* < .001).

Results are presented in Figures 4 and 5. A 2 × 6 × 2 repeated measures ANOVA was used to analyse percentage viewing time in which setting direction (backward, forward), fixation locations (coach, legs, hands, out, ball, and trunk), and response accuracy (correct, incorrect) were the within-subjects factors and expertise (experts, novices) the between-subjects factors. The analysis indicated significant main effects for expertise (*F*_1,28_ = 3.84, *P* = .048, and *ηp* ^2^ = .06), setting direction (*F*_1,28_ = 10.73, *P* = .003, and *ηp* ^2^ = .27), fixation locations (*F*_5,140_ = 17.20, *P* < .001, and *ηp* ^2^ = .38), and accuracy (*F*_1,28_ = 27.54, *P* < .001, and *ηp* ^2^ = .49). Analysis also showed expertise × accuracy (*F*_1,28_ = 12.41, *P* < .001, and *ηp* ^2^ = .30) and fixation locations × accuracy × expertise (*F*_5,140_ = 9.46, *P* < .001, *ηp* ^2^ = .25) interactions.* t*-tests, with Bonferroni correction, revealed that when the responses were incorrect, experts showed longer fixations on legs [*t*(28) = 5.28; *P* < .001] and on hands [*t*(28) = 3.40; *P* < .001] areas in comparison to novices (see Figure 4(a)).

Paired sample* t*-tests, with Bonferroni correction, showed that only experts revealed significant differences between areas on correct/incorrect trials, looking longer on legs [*t*(14) = 9.13; *P* < .001], hands [*t*(14) = 5.70; *P* < .001], and trunk [*t*(14) = 3.95; *P* < .001] areas when the responses were incorrect than correct (see Figure 5(a) (right panel)). No significant differences were found for the novice group (see Figure 5).

In correct responses, experts spent less time fixating on all locations with respect to novices and more time fixating on leg and hand areas in incorrect ones. The percentage of time spent by experts increased during backward setting and when they made wrong responses (see Figure 5).

## 4. Discussion

The aims of this study were (1) to examine the relationships between visual search behaviour and anticipatory responses in order to discover differences between expert and novice volleyball players and (2) to compare gaze strategies and action effectiveness in an expert group.

As predicted, experts had better performance on the anticipation test than their novice counterpart, in that they were more accurate in predicting the direction of the setting and faster in response than novices' group. They showed 91% of correct responses against 77% of novices and a shorter key-press response time. In the gaze behaviour domain, experts, as already shown in a previous study [13], used a search strategy involving less number of fixations of longer duration than their novice counterpart.

As was mentioned in previous reports [4, 32], experts are able to reduce the amount of information to be processed or require fewer fixations to create a coherent perceptual representation of the display. Our results seem to be in contrast with the theory of long-term working memory [33], for which experts when encode and retrieve information more rapidly than novices need shorter instead of longer fixation durations. On the other hand, our results on fixation durations are in agreement with the information reduction hypothesis, in which experts should exhibit longer fixation durations on task-relevant areas [25]. Our experts demonstrated longer fixation durations on task-relevant areas, especially when time on task was limited and response accuracy was a performance predictor. Moreover, we found significant correlations between fixation durations and number of fixations in experts and novices' correct responses and between fixation durations and response time in expert's correct response, when fixation number was a control variable. Results indicate that as the number of fixations decreases, duration of each fixation increases linearly only when the responses are correct. This is in line with the simple heuristics explanation and previous studies that reported that experts show less fixations of longer duration within the task [22]. Experts also showed longer fixations when response time decreased and they gave correct responses, which resulted in superior performance, characterized by faster decision times and greater response accuracy. This is interpreted as a simple heuristic and is in line with the hypothesis that athletes use less information or require fewer cognitive steps to arrive at a correct prediction. From this perspective, search is based on the importance of the cues and their reciprocal relationships. If one cue is more important than the sum of the others, experts can use this knowledge, which they gain from experience, and stop searching after considering this cue. As a matter of fact, the mean percentual viewing time analysis of incorrect responses, in this research, was similar to that of our previous paper, where athletes fixated the gaze particularly on hands, trunk, and legs of the setter [13]. In the present experiment, athletes, instructed to anticipate rather than just observe film clips, fixated longer on legs, hands, and trunk in incorrect than in correct trials. The fixation of setter's body parts (legs, trunk, and hands) by the experts could be explained by the fact that they chose to anchor the fovea close to these key locations so that they could use the parafovea and visual periphery to pick up relevant information [27]. The effective use of such “visual pivots,” in which the gaze is centrally located between locations, thus enabling the optimal use of both the foveal and parafoveal vision, suggests that a specific information cue is less important than the relative motions between these areas (i.e., legs, hands, and trunk). A visual pivot is needed when spatial information is complex and where there is a need to shift the gaze quickly between different locations [34]. We would agree and add that, in volleyball, experts use such visual pivot for maximum information extraction under extreme time constraints, and simultaneously, we can assume that if they remain for too long time there, the likelihood to respond incorrectly increases. Moreover, when the comparison was done between groups, experts revealed more fixations on legs and hands areas in incorrect responses. It seems that setter's hands, legs, and trunk, if fixated for too long time, are not the optimal areas to predict the future setting direction.

The results did not support all predictions of the information reduction hypothesis, as errors, or poorer performance, could not be related to longer inspection of task-irrelevant areas. Although both recognition (our first study) and anticipation (present results) tasks stimulate complex retrieval structures, the processes involved in activating these structures do differ to some degree. While recognition may be involved in anticipation, the latter expertise appears more complex, invoking different and more refined retrieval structures [35, 36]. Present results suggest that, for experts, more time spent in fixating on legs, hands, and trunk ended up with more incorrect than correct responses. In fact, experts' group spent more time than novices on all interest areas when they gave incorrect responses and less time when the responses were correct. Considering just experts' group, they spent longer period of time fixating on legs, hands, and trunk when they gave wrong responses during backward setting. It seems that athletes, trying to foresee setter's intention, watch longer all interest areas. This could be because setter's actions are more difficult in a subset of videos and the increased difficulty level brings about the divergent eye movement patterns as well as the wrong responses. This might be related to the difficulty to “catch visual cues” from the setter when time is short. Volleyball players often have to quickly shift from a diffuse attention, coarsely attending to opponent's scene, to a more focused attention, aiming at opponent's body or at the ball. Results of visual scanning studies and kinematics analysis of volleyball settings show that expert players direct their gaze not only towards specific relevant information in the visual field, but also towards intermediate positions between several visual cues [34]. Thus, it can be assumed that volleyball players perform zooming operations, adapting the span of the attentional focus to encompass only few or many elements depending on the game context.

In conclusion, the present study discerned two strategies among expert players in their attempts to block two different types of volleyball attack. With respect to setting directions, experts appear to use a different strategy in backward than forward settings. In fact, in the first case, they watched for too long all interest areas, especially legs, hands, and trunk, while, in forward setting, they watched equally, for short times, coach, ball, hands, and trunk areas and, more, although not significant, setter's legs. With respect to response accuracy, experts employ a distinct anticipation strategy in correct responses, where they watched equally, for short times, all areas, while in incorrect responses they watched more the setter's legs, hands, and trunk. The critical difference for success seems not to fix for long time any of the interest areas to decide the ball destination but to use a visual search strategy aimed at the most efficient extraction of information per fixation. The best parameters for successful performance, on expert group, were few number of fixations, of 500 ms each, during the phase before hands-ball contact, an anticipatory response time between 346 and 367 ms after the hands-ball contact, and a low number of interest areas fixated per trial.

Our study confirmed the superior speed of information processing achieved by experts and their greater accuracy in task performance than novice players. Moreover, these findings imply that experts are able to extract relevant information from different body areas simultaneously when attempting to anticipate their opponents' intentions [35]. Expert athletes optimize the amount of processed information focusing on task-relevant information and selectively allocating attentional resources.

## Figures and Tables

**Table 1:** Dependent measures, choice response time (CRT) and response accuracy (RA), recorded on the anticipation test across groups (mean ± *s*). Only trials with response time between 150 and 600 msec are reported.

			Experts	Novices
		Total	351.02 ± 14.47	406.21 ± 12.19
Choice reaction time (ms)		Correct response	385.31 ± 21.23	397.48 ± 17.72
		Incorrect response	316.73 ± 17.91	414.94 ± 16.91

		Incorrect	92.00	154.00
	Total	Correct	888.00	515.00
		Total count	980.00	669.00
		% of total forward	88.00%	72.00%
Response accuracy (number of trials)	Correct	% of total backward	93.00%	81.00%
		% of total	91.00%	77.00%
		% of total forward	12.00%	28.00%
	Incorrect	% of total backward	7.00%	19.00%
		% of total	9.00%	23.00%
